# Acute Kidney Injury in Pregnancy: A Prospective Study

**DOI:** 10.7759/cureus.58982

**Published:** 2024-04-25

**Authors:** Manoj Kumar Choudhary, Arshad Ahmad, Anuradha Kumari, Dipali Prasad, Naresh Kumar

**Affiliations:** 1 General Medicine, Indira Gandhi Institute of Medical Sciences, Patna, IND; 2 Obstetrics and Gynecology, Indira Gandhi Institute of Medical Sciences, Patna, IND; 3 Medicine, Indira Gandhi Institute of Medical Sciences, Patna, IND

**Keywords:** maternal outcome, hemodialysis, sepsis, acute kidney injury, pregnancy

## Abstract

Introduction: When acute kidney damage occurs during pregnancy, it poses a difficult clinical problem. One of the main causes of maternal and fetal morbidity and death is pregnancy-related acute kidney injury (AKI), a significant obstetric complication characterized by a fast deterioration in renal function and several subsequent clinical problems. The objective of the study is to analyze the etiological factors, clinical manifestations, and maternal and fetal outcomes of AKI during pregnancy.

Materials and methods: This prospective observational research involved patients hospitalized in the General Medicine and Obstetrics and Gynecology departments at Indira Gandhi Institute of Medical Sciences, Patna, for a year (October 2021 to September 2022) due to obstetric difficulties resulting in acute renal damage.

Results: The study included 62 patients with a mean age of 25.08±4.25 years. The majority of patients in our study were aged 18-25 years (38, 61.3%), followed by 26-30 years (19, 30.6%) and >30 years (5, 8.1%). The majority of patients in our study were non-booked (52, 83.9%) and presented as emergency cases, whereas 10 (16.1%) patients had booked. In addition, 34 (54.8%) patients were primigravida, while 28 (45.1%) were multigravida. There were 25 patients in their third trimester (40.3%), 19 who were postpartum (30.6%), 10 who were post-abortion (16.1%), and eight in their second trimester (12.9%). Upon admission, the majority of the patients showed signs of oliguria, accounting for 45 cases (72.6%). This was followed by nine cases of abnormal kidney function (14.5%) and eight cases of anuria (12.9%). Among the other symptoms, fever was observed in 25 cases (40.32%), whereas breathlessness increased to 15 cases (24.19%), edema was present in 14 cases (22.58%), vomiting and altered sensorium were observed in four cases (6.45%), abdominal pain was observed in three cases (4.83%), and burning micturition was observed in two cases (3.22%). The most common causes of AKI in pregnancy in the present study were puerperal sepsis (18 cases, 29.0%), followed by preeclampsia/eclampsia (14 cases, 22.6%), hemorrhagic shock (10 cases, 16.1%), septic abortion (six cases, 9.7%), hyperemesis gravidarum (four cases, 6.5%), acute fatty liver of pregnancy (three cases, 4.8%), disseminated intravascular coagulation (three cases, 4.8%), drug-induced sepsis (two cases, 3.2%), and urosepsis (two cases, 3.2%). Modes of delivery in this study were normal vaginal delivery (32 cases, 51.6%), lower segment cesarean section (21 cases, 33.9%), dilation and evacuation (seven cases, 11.3%), and total hysterectomy (two cases, 3.2%). Hemodialysis was performed in 39 patients (62.9%), and 51 (82.3%) received blood transfusions. The mean systolic and diastolic BP (mmHg) were 111.37±22.60 and 71.40±18.88, respectively. Maternal outcome data revealed that 48 (77.4%) women had fully recovered, eight (12.9%) had not recovered, 43 (69.4%) were lost to follow-up, and two (3.2%) had died. Neonatal outcomes in the present study were as follows: live birth, 43 (69.4%); abortion, eight (12.9%); intrauterine death of the fetus, five (8.1%); and neonatal mortality, six (9.7%).

Conclusion: The diagnosis and treatment of AKI during pregnancy is a significant challenge for the treating physician because of the pathophysiological changes that occur during pregnancy, the variability of symptoms, and the fact that clinical and laboratory features may occasionally overlap.

## Introduction

One major cause of maternal and fetal morbidity and death during pregnancy is acute kidney injury (AKI), which is a public health concern [1]. There is an increase in the risk of hospitalization and long-term mortality from AKI [2]. In addition, AKI worsens kidney function, which leads to chronic kidney disease and end-stage kidney disease, which in turn increases the risk of unfavorable cardiovascular events, healthcare use, and hospital stays [3,4]. Hence, it is crucial to identify and treat AKI in pregnancy as soon as possible; doing so might save lives. Legalizing abortion and improving prenatal and obstetric care have led to a dramatic decline in the incidence of pregnancy-related acute kidney damage during the last half century, from 20-40% in 1960 to less than 10% in the present series.

There is a significant risk of maternal death and fetal loss ranging from 30% to 60% linked to AKI during pregnancy (PR-AKI), which is a potentially fatal condition [5]. According to the Kidney Disease Improving Global Outcomes definition, AKI is characterized as follows: a serum creatinine level greater than 0.3 mg/dL from baseline or more than 1.5 times the reference value or a decrease in urine output below 0.5 mL/kg/hour lasting 6-12 hours. The purpose of the study is to identify the current trend in PR-AKI. This study was a prospective observational study. Its objective is to analyze the etiological factors, clinical manifestations, and maternal and fetal outcomes of PR-AKI.

## Materials and methods

Patient selection

Patients hospitalized in the General Medicine and Obstetrics and Gynecology departments at Indira Gandhi Institute of Medical Sciences, Patna, for a year, from October 2021 to September 2022, with obstetric difficulties resulting in acute renal damage, were the participants of this prospective observational study. The study received ethical clearance from the Institutional Ethics Committee of Indira Gandhi Institute of Medical Sciences, Patna (approval number: 1791/IEC/IGIMS/2020, dated September 30, 2020). This study included pregnant women who were previously healthy and had developed AKI, who had an increase in serum creatinine ≥0.3 mg/dl within 48 hours, who had an increase in serum creatinine ≥1.5 times from baseline, who had an oliguria urine output <0.5 ml/kg/h for six hours, and who had given consent for the study. The study excluded patients with evidence of renal disease before pregnancy, a history of renal stone diseases, and elevated serum creatinine before gestation.

Outcome of interest

We analyzed patients by examining their demographics, medical history, clinical symptoms, and results from several laboratory tests (e.g., complete blood count, prothrombin time/international normalized ratio (PT/INR), liver function test, kidney function test, and ultrasound of the whole abdomen). A comprehensive obstetric evaluation was performed on every patient. We asked detailed questions about the birth method, blood transfusion, and surgical procedure and whether dialysis was necessary. Reversible renal failure, partial recovery, and full recovery were the metrics used to measure maternal outcomes. Standard indications were followed when hemolysis was conducted. When the patient was able to stop using dialysis and maintain normal renal function and urine production, it was deemed a positive result, representing a full recovery. Partial recovery was defined as patients who were dialysis-independent upon discharge and whose renal function had improved, but not to a normal level. An undesirable result was the onset of chronic renal disease or death. Chronic renal disease was diagnosed in patients when dialysis was still necessary three months after discharge.

Statistical analysis

We encrypted and stored all the data in MS Excel. We analyzed the obtained data using IBM SPSS Statistics for Windows, Version 23.0 (Released 2015; IBM Corp., Armonk, New York, United States) for this investigation. For continuous variables, the descriptive statistics were given as medians, interquartile ranges, and standard deviations; for categorical variables, the frequencies, percentages, and ages were given. To compare two groups utilizing continuously dispersed data, the t-test for independent samples was used. Data that did not conform to a normal distribution were subjected to the Wilcoxon test or other suitable nonparametric tests. We developed the chi-squared test to compare sets of category data thoroughly. Since the predicted frequency was less than five, Fisher's exact test was used for over 25% of the cells in the contingency tables. When the p-value was less than 0.05 or 0.001, the findings were deemed significant.

## Results

 A total of 62 patients were included in the present study. The age of the patients in this study ranged from 18 to 40 years, with a mean age of 25.08±4.25 years and a median age of 24.50 (22.00-26.75) years. The majority of patients in our study were aged 18-25 years (38, 61.3%) followed by 26-30 years (19, 30.6%) and >30 years (5, 8.1%). Of the 62 patients, 31 (50.0%) were of middle socioeconomic status, followed by 23 of low socioeconomic status (37.1%) and eight of upper socioeconomic status (12.9%). The majority of patients in our study were non-booked (52, 83.9%) and presented as emergency cases, whereas 10 (16.1%) patients were booked. The majority of patients in this study were primigravida (34, 54.8%), followed by multigravida (28, 45.1%). The majority of the patients were in the third trimester (25, 40.3%), followed by postpartum (19, 30.6%), post-abortion (10, 16.1%), and patients in the second trimester (8, 12.9%). At admission, patients most commonly presented with oliguria (45, 72.6%), abnormal kidney function (9, 14.5%), and anuria (8, 12.9%). The most common cause of AKI in pregnancy in the present study was puerperal sepsis (18, 29.0%), followed by preeclampsia/eclampsia (14, 22.6%), hemorrhagic shock (10, 16.1%), septic abortion (6, 9.7%), hyperemesis gravidarum (4, 6.5%), acute fatty liver of pregnancy (AFLP) (3, 4.8%), disseminated intravascular coagulation (DIC) (2, 3.2%), drug-induced sepsis (2, 3.2%), and urosepsis (2, 3.2%). The modes of delivery in this study were normal vaginal delivery (32, 51.6%), lower segment cesarean section (21, 33.9%), dilation and evacuation (7, 11.3%), and total hysterectomy (2, 3.2%). Hemodialysis was done in 39 (62.9%) of the patients. Blood transfusions were performed in 51 (82.3%) patients. The mean systolic BP (mmHg) was 111.37±22.60, and the mean diastolic BP (mmHg) was 71.40±18.88 (Table 1).

**Table 1:** Summary of clinical details Data are shown as N (%) N (%): number (percentage); DIC: disseminated intravascular coagulation; AFLP: acute fatty liver of pregnancy; KFT: kidney function test; AKI: acute kidney injury

Clinical details	N (%)
Age group (years)	
18-25	38 (61.3%)
26-30	19 (30.6%)
>30	5 (8.1%)
Socioeconomic status	
Lower	23 (37.1%)
Middle	31 (50.0%)
Upper	8 (12.9%)
Booking status	
Booked	10 (16.1%)
Non-booked	52 (83.9%)
Obstetric history	
Primigravida	34 (54.8%)
Multigravida	28 (45.1%)
Stage of pregnancy	
Second trimester	8 (12.9%)
Third trimester	25 (40.3%)
Post-abortion	10 (16.1%)
Postpartum	19 (30.6%)
Clinical presentation at the time of admission	
Abnormal KFT	9 (14.5%)
Oliguria	45 (72.6%)
Anuria	8 (12.9%)
Etiology of AKI	
Preeclampsia/eclampsia	14 (22.6%)
Hemorrhagic shock	10 (16.1%)
DIC	3 (4.8%)
Puerperal sepsis	18 (29.0%)
Septic abortion	6 (9.7%)
AFLP	3 (4.8%)
Hyperemesis gravidarum	4 (6.5%)
Drug-induced sepsis	2 (3.2%)
Urosepsis	2 (3.2%)
Mode of delivery	
Normal vaginal delivery	32 (51.6%)
Lower segment cesarean section	21 (33.9%)
Dilatation and evacuation	7 (11.3%)
Total hysterectomy	2 (3.2%)
Hemodialysis (yes)	39 (62.9%)
Blood transfusion (yes)	51 (82.3%)

Upon admission, all 62 women exhibited significantly elevated levels of serum creatinine and blood urea nitrogen, with a mean hemoglobin percentage of 7.99±1.43. The median hemoglobin level was 5.80 (ranging from 3.85 to 6.97), with extremes ranging from 1.50 to 12.00. Additionally, the mean total leukocyte count (TLC) stood at 18075.97±17026.29 cells/mm³, with a median of 15790.00 (ranging from 12000.00 to 19637.50) and an overall range of 5100.00-142000.00. Liver function tests revealed mean alanine transaminase/aspartate aminotransferase (ALT/AST) levels of 99.19±44.02 and 80.56±23.30 U/L, respectively, with median values of 80.00 (ranging from 70.00 to 117.50) and 78.00 (ranging from 70.00 to 90.00). The range for ALT/AST was 37.00-230.00 and 50.00-220.00, respectively. Furthermore, the mean serum sodium/serum potassium levels were 132.77±3.91 and 4.84±0.98 mEq/L, with median values of 132.00 (ranging from 129.25 to 135.00) and 4.90 (ranging from 4.10 to 5.60). The respective ranges were 128.00-146.00 and 2.90-6.50. Serum albumin levels averaged 2.99±0.53 gm/dl, with a median of 3.00 (ranging from 2.52 to 3.40). The range for serum albumin was 2.10-4.00. Prothrombin time (PT)/INR averaged 17.31±2.68 and 1.46±0.28 seconds, with median values of 17.00 (ranging from 15.25 to 18.75) and 1.50 (ranging from 1.22 to 1.60) seconds. The range for PT/INR was 11.00-24.00 and 1.00-2.30, respectively. Additionally, mean fibrin degradation product (FDP) levels were 276.34±69.00 µg/mL, with a median of 285.50 (ranging from 236.50 to 336.00) and an overall range of 149.00-400.00. The detailed biochemical parameters are presented in Table 2.

**Table 2:** Summary of investigations Data are shown as mean±SD, median (IQR), and min-max range SD: standard deviation; IQR: interquartile range; TLC: total leukocyte count; ALT: alanine transaminase; AST: aspartate aminotransferase; PT: prothrombin time, INR: international normalized ratio; FDP: fibrinogen degradation products

Investigations	Mean±SD	Median (IQR)	Min-max
Creatinine (mg/dl) (admission)	5.52±2.23	5.80 (3.85-6.97)	1.50-12.00
BUN (mg/dl) (admission)	50.74±27.65	44.00 (29.75-60.00)	12.60-135.00
Hemoglobin (g/dL)	7.99±1.43	8.00 (7.00-9.00)	4.40-12.90
TLC (cells/mm³)	18075.97±17026.29	15790.00 (12000.00-19637.50)	5100.00-142000.00
ALT (U/L)	99.19±44.02	80.00 (70.00-117.50)	37.00-230.00
AST (U/L)	80.56±23.30	78.00 (70.00-90.00)	50.00-220.00
LDH (U/L)	596.89±657.68	347.50 (244.25-497.25)	156.00-3200.00
Serum sodium (mEq/L)	132.77±3.91	132.00 (129.25-135.00)	128.00-146.00
Serum potassium (mEq/L)	4.84±0.98	4.90 (4.10-5.60)	2.90-6.50
Serum albumin (gm/dl)	2.99±0.53	3.00 (2.52-3.40)	2.10-4.00
PT (seconds)	17.31±2.68	17.00 (15.25-18.75)	11.00-24.00
INR (seconds)	1.46±0.28	1.50 (1.22-1.60)	1.00-2.30
FDP (µg/mL)	276.34±69.00	285.50 (236.50-336.00)	149.00-400.00

The most common cause of AKI in pregnancy in the present study was puerperal sepsis (18, 29.0%), followed by preeclampsia/eclampsia (14, 22.6%), hemorrhagic shock (10, 16.1%), septic abortion (6, 9.7%), hyperemesis gravidarum (4, 6.5%), AFLP (3, 4.8%), DIC (3, 4.8%), drug-induced AKI (2, 3.2%), and urosepsis (2, 3.2%) (Table 3).

**Table 3:** Distribution of participants in terms of "etiology of AKI" Data are shown as frequency, percentage, and 95% CI CI: confidence interval; AFLP: acute fatty liver of pregnancy; AKI: acute kidney injury

Etiology of AKI	Frequency	Percentage	95% CI
Preeclampsia/eclampsia	14	22.6%	13.3-35.3%
Hemorrhagic shock	10	16.1%	8.4-28.1%
DIC	3	4.8%	1.3-14.4%
Puerperal sepsis	18	29.0%	18.6-42.1%
Septic abortion	6	9.7%	4.0-20.5%
AFLP	3	4.8%	1.3-14.4%
Hyperemesis gravidarum	4	6.5%	2.1-16.5%
Drug-induced AKI	2	3.2%	0.6-12.2%
Urosepsis	2	3.2%	0.6-12.2%

Fisher's exact test was used to explore the association between the etiology of AKI and symptoms at presentation, as more than 20% of the total number of cells had an expected count of less than 5. There was a significant difference between the various groups in terms of the distribution of symptom at presentation (χ2=50.438; p<0.001 is considered significant). At admission, patients most commonly presented with oliguria (45, 72.6%), followed by abnormal kidney function (9, 15.5%) and anuria (8, 12.9%) (Table 4).

**Table 4:** Association between the etiology of AKI and symptoms at presentation Data are shown as χ2-Fisher's exact test; p<0.001 is considered statistically significant

Symptom at presentation	Etiology of AKI										Fisher's exact test	
	Preeclampsia/eclampsia	Hemorrhagic shock	DIC	Puerperal sepsis	Septic abortion	AFLP	Hyperemesis gravidarum	Drug-induced AKI	Urosepsis	Total	χ2	P-value
Abnormal KFT	0 (0.0%)	4 (40.0%)	0 (0.0%)	0 (0.0%)	0 (0.0%)	0 (0.0%)	3 (75.0%)	0 (0.0%)	2 (100.0%)	9 (14.5%)	50.438	<0.001
Oliguria	8 (57.1%)	6 (60.0%)	3 (100.0%)	16 (88.9%)	6 (100.0%)	3 (100.0%)	1 (25.0%)	2 (100.0%)	0 (0.0%)	45 (72.6%)		
Anuria	6 (42.9%)	0 (0.0%)	0 (0.0%)	2 (11.1%)	0 (0.0%)	0 (0.0%)	0 (0.0%)	0 (0.0%)	0 (0.0%)	8 (12.9%)		
Total	14 (100.0%)	10 (100.0%)	3 (100.0%)	18 (100.0%)	6 (100.0%)	3 (100.0%)	4 (100.0%)	2 (100.0%)	2 (100.0%)	62 (100.0%)		

**Figure 1:**
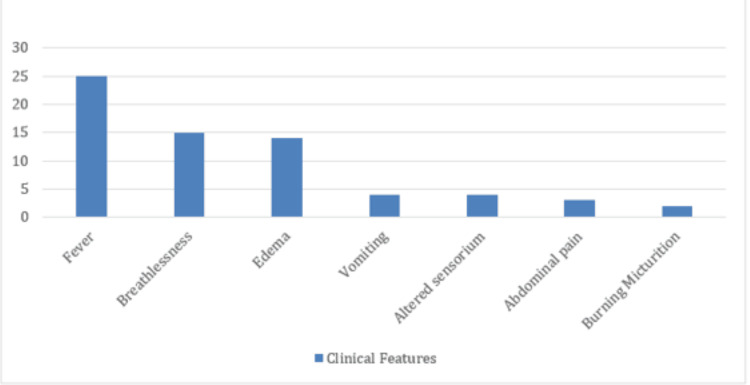
Clinical feature of the patients Data is shown as a bar graph representing clinical features on the x-axis and the number of patients on the y-axis

As depicted in Figure 1, patients presented with fever (25, 40.32%), followed by breathlessness (15, 24.19%), edema (14, 22.58%), vomiting (4, 6.65%), altered sensorium (4, 6.45%), abdominal pain (3, 4.83%), and burning micturition (2, 3.22%).

Fisher's exact test was used to explore the association between the etiology of AKI and maternal outcomes, as more than 20% of the total number of cells had an expected count of less than 5. There was a significant difference between the various groups in terms of the distribution of maternal outcomes (χ2=64.399, p<0.001). Maternal outcome data showed that 48 (77.4%) women had fully recovered, eight (12.9%) had not recovered, four (6.5%) were lost to follow-up, and two (3.2%) had died (Table 5).

**Table 5:** Association between the etiology of AKI and maternal outcomes Data are shown as χ2-Fisher's exact test; p<0.001 is considered statistically significant

Maternal outcome	Etiology of AKI										Fisher's exact test	
	Preeclampsia/eclampsia	Hemorrhagic shock	DIC	Puerperal sepsis	Septic abortion	AFLP	Hyperemesis gravidarum	Drug-induced AKI	Urosepsis	Total	χ2	P-value
Complete recovery	11 (78.6%)	10 (100.0%)	2 (66.7%)	15 (83.3%)	0 (0.0%)	2 (66.7%)	4 (100.0%)	2 (100.0%)	2 (100.0%)	48 (77.4%)	64.399	<0.001
Unrecovered	0 (0.0%)	0 (0.0%)	1 (33.3%)	1 (5.6%)	6 (100.0%)	0 (0.0%)	0 (0.0%)	0 (0.0%)	0 (0.0%)	8 (12.9%)		
Lost to follow-up	3 (21.4%)	0 (0.0%)	0 (0.0%)	1 (5.6%)	0 (0.0%)	0 (0.0%)	0 (0.0%)	0 (0.0%)	0 (0.0%)	4 (6.5%)		
Death	0 (0.0%)	0 (0.0%)	0 (0.0%)	1 (5.6%)	0 (0.0%)	1 (33.3%)	0 (0.0%)	0 (0.0%)	0 (0.0%)	2 (3.2%)		
Total	14 (100.0%)	10 (100.0%)	3 (100.0%)	18 (100.0%)	6 (100.0%)	3 (100.0%)	4 (100.0%)	2 (100.0%)	2 (100.0%)	62 (100.0%)		

**Figure 2:**
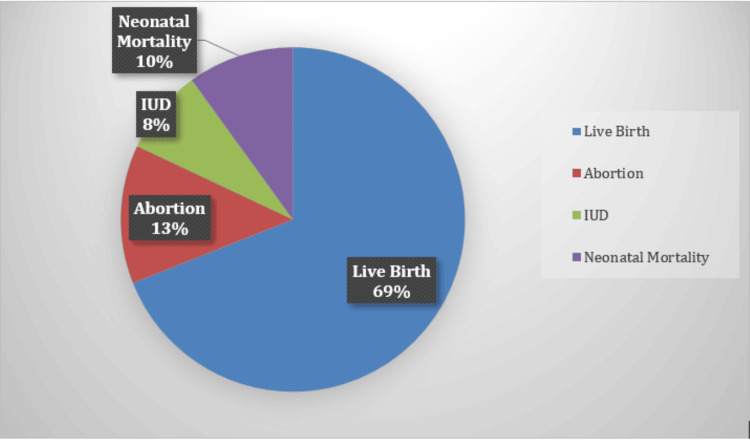
Distribution of fetal outcome IUD: intrauterine death

Figure 2 illustrates the neonatal outcomes: 43 births (69.4%), eight abortions (12.9%), five IUDs (8.1%), and six neonatal deaths (9.7%).

Table 6 shows that 39 (69.2%) of patients diagnosed with AKI were managed conservatively without hemodialysis, whereas 23 (37.1%) of patients needed hemodialysis. 

**Table 6:** Distribution of the participants in terms of "hemodialysis" Data are shown as frequency, percentage, and 95% CI CI: confidence interval

Hemodialysis	Frequency	Percentage	95% CI
Yes	39	62.9%	49.7-74.6%
No	23	37.1%	25.4- 50.3%

**Figure 3:**
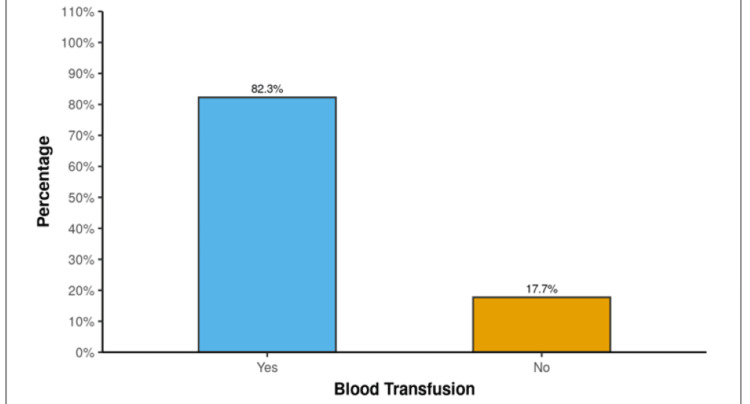
Distribution of blood transfusion

Figure 3 shows that 51 (82.3%) patients required a blood transfusion, whereas 11 (17.7%) did not. 

## Discussion

Obstetric AKI has a significant influence on both fetal and maternal complications [6]. The literature has reported the incidence of AKI during pregnancy as 1-2.8% for developed countries, whereas in developing countries, it is almost 9-25.3% [7,8]. The incidence is lower in developed countries because of appropriate antenatal care, timely diagnosis, and treatment of complications [9]. The age range in our study was 18-40 years, with a mean age of 25.08±4.25. A study conducted by Arrayhani et al. [6] reported an age range of 18-40 years. In a study by Verma and Sharma [10], the mean age was 29.33±5.31 years, and it was 25.23±3.8 years in a study by Paudyal et al. [11]. The mean age was almost the same in all related studies. This might be because all the studies focused on AKI related to pregnancy and the mean age of marriage and pregnancy is almost identical among most young women.

The majority of patients in our study were non-booked patients (52, 83.9%) and presented as emergency cases. Only 10 (16.1%) patients in our study received regular antenatal care. A study conducted by Mehta et al. [12] also reported that 66% of the patients in their study were not booked and did not receive adequate antenatal care. The most common etiological factor for the development of AKI in our study was puerperal sepsis (18 cases, 29.0%), followed by preeclampsia/eclampsia (14 cases, 22.6%), hemorrhagic shock (10 cases, 16.1%), septic abortion (six cases, 9.7%), hyperemesis gravidarum (four cases, 6.5%), DIC (three cases, 4.8%), AFLP (three cases, 4.8%), urosepsis (two cases, 3.2%), and drug-induced AKI (two cases, 3.2%). The drugs responsible for AKI in this study were amikacin and diclofenac sodium.

Sepsis was the main cause of AKI in a study conducted by Mahesh et al. [13], in contrast to a study conducted by Taj et al., which identified postpartum hemorrhage (eight cases, 18.2%) as the main cause [14], causing hypotension for the development of AKI in pregnancy. Preeclampsia/eclampsia (14 cases, 26.6%) is another common cause of AKI in our study, which is consistent with the study by Prakash et al., which reported preeclampsia and eclampsia to be the most common causes of pregnancy-related acute renal injury, contributing to 35.29% of cases [15].

Huang and Chen also identified preeclampsia/eclampsia as the common etiological factor for the occurrence of AKI in pregnancy [16]. Patients with AKI may have clear symptoms at presentation. The most common presentation at the time of diagnosis of AKI in our study was oliguria (42 cases, 72.6%). Oliguria was also the most common presentation in the studies conducted by other authors, such as Gayathiri et al., who reported that 57% of patients had oliguria [17]. In this study, 23 (69.2%) patients diagnosed with AKI were managed conservatively without hemodialysis, whereas 39 (37.1%) patients needed hemodialysis. In a study by Paudyal et al., the need for hemodialysis was as high as 80% [11], and in a study conducted by Gayathiri et al., two (33%) patients required hemodialysis, and 11% required plasmapheresis [18]. The lack of proper emergency services in the periphery hospitals and late diagnosis of disease and referral may be directly related to the need for hemodialysis. This is a common problem in developing countries due to inadequate infrastructure. Complete recovery was seen in 48 (77.4%) patients in our study. In a study conducted in Morocco by Arrayhani et al., complete recovery was seen in 75.67% of the patients [6], and according to the study conducted by Trakarnvanich et al. [19], 70.6% of patients with PR-AKI achieved complete recovery, 14.7% achieved partial recovery, and 8.5% remained dependent on dialysis. The limitation of the present study is that there was a short duration of follow-up, as further long-term follow-up is required to assess the outcome of patients with chronic kidney disease.

## Conclusions

Acute kidney damage poses a difficult clinical problem when it occurs during pregnancy. AKI during pregnancy continues to be a major public health problem in underdeveloped nations. In the present study, the leading cause of AKI was sepsis, which became worse throughout pregnancy. In addition to sepsis, which is both curable and preventive, postpartum hemorrhage and antepartum hemorrhage were the leading causes of AKI. Deaths and illnesses caused by AKI could be reduced with improved prenatal care, easier access to emergency care, and prompt diagnosis and referral.
